# Radiomics nomogram for preoperative differentiation of pulmonary mucinous adenocarcinoma from tuberculoma in solitary pulmonary solid nodules

**DOI:** 10.1186/s12885-023-10734-4

**Published:** 2023-03-21

**Authors:** Junjie Zhang, Ligang Hao, MingWei Qi, Qian Xu, Ning Zhang, Hui Feng, Gaofeng Shi

**Affiliations:** 1 Department of Computed Tomography and Magnetic Resonance, Hebei Medical University Fourth Affiliated Hospital, 12 Jiankang Road, Shijiazhuang, 050011 Hebei China; 2 Department of CT&MR, The First Hospital of Xing Tai, Xing Tai, 054000 He Bei China; 3 Department of Thoracic Surgery Xing, Tai People’s Hospital, Xing Tai, 054000 He Bei China

**Keywords:** Radiomics, Contrast-enhanced CT, Mucinous adenocarcinoma, Tuberculoma, Nomogram

## Abstract

**Objective:**

To develop and validate predictive models using clinical parameters, radiomic features and a combination of both for preoperative differentiation of pulmonary nodular mucinous adenocarcinoma (PNMA) from pulmonary tuberculoma (PTB).

**Method:**

A total of 124 and 53 patients with PNMA and PTB, respectively, were retrospectively analyzed from January 2017 to November 2022 in The Fourth Affiliated Hospital of Hebei Medical University (Ligang et al., A machine learning model based on CT and clinical features to distinguish pulmonary nodular mucinous adenocarcinoma from tuberculoma, 2023). A total of 1037 radiomic features were extracted from contrast-enhanced computed tomography (CT). The patients were randomly divided into a training group and a test group at a ratio of 7:3. The least absolute shrinkage and selection operator (LASSO) algorithm was used for radiomic feature selection. Three radiomics prediction models were applied: logistic regression (LR), support vector machine (SVM) and random forest (RF). The best performing model was adopted, and the radiomics score (Radscore) was then computed. The clinical model was developed using logistic regression. Finally, a combined model was established based on clinical factors and radiomics features. We externally validated the three models in a group of 68 patients (46 and 22 patients with PNMA and PTB, respectively) from Xing Tai People’s Hospital (30 and 14 patients with PNMA and PTB, respectively) and The First Hospital of Xing Tai (16 and 8 patients with PNMA and PTB, respectively). The area under the receiver operating characteristic (ROC) curve (AUC) value and decision curve analysis were used to evaluate the predictive value of the developed models.

**Results:**

The combined model established by the logistic regression method had the best performance. The ROC-AUC (also a decision curve analysis) of the combined model was 0.940, 0.990 and 0.960 in the training group, test group and external validation group, respectively, and the combined model showed good predictive performance for the differentiation of PNMA from PTB. The Brier scores of the combined model were 0.132 and 0.068 in the training group and test group, respectively.

**Conclusion:**

The combined model incorporating radiomics features and clinical parameters may have potential value for the preoperative differentiation of PNMA from PTB.

**Supplementary Information:**

The online version contains supplementary material available at 10.1186/s12885-023-10734-4.

## Introduction

Lung cancer is the leading cause of cancer-related deaths worldwide and one of the most common malignancies in China [1, 2]. In primary lung cancer, non-small cell lung cancer (NSCLC) accounts for almost 85% of the cases, of which adenocarcinoma is the most frequent pathological subtype [3, 4]. According to the International Association for the Study of Lung Cancer (IASLC)/American Thoracic Society (ATS)/European Respiratory Society (ERS) classification system for lung adenocarcinoma published in 2011, as well as the World Health Organization (WHO) published in 2015, primary pulmonary invasive mucinous adenocarcinoma (IMA) was classified as a variant subtype of lung adenocarcinoma, accounting for 2–5% of the cases of adenocarcinoma [5, 6].

Previous studies have explored the imaging findings of IMA [7, 8]. On the basis of CT findings, IMA is mainly divided into two types: pulmonary nodule-type IMA (PNMA) and pneumonia-type IMA [9, 10]. PNMA nodules, especially nodules with ill-defined margins, internal cavities or vacuoles, and no-mild enhancement on dynamic CT, were similar to benign solid nodules. Pulmonary tuberculoma (PTB) is the most common type of benign solid nodule [11]. The CT characteristics of PTB can also include spiculated signs and pleural indentation, which can make it difficult to distinguish PTB from PNMA. There may be overlap in the morphology and CT features between PNMA and PTB. In fact, many cases of PNMA are misdiagnosed as PTB in clinical practice, which leads to missing the best treatment time, leading to a shortened survival time [12, 13]. Distinguishing PNMA and PTB before treatment in a timely manner is important.

Computed tomography (CT) is widely used for tumor detection, staging and therapeutic response monitoring in clinical practice [14]. There have been few studies concerning the differential diagnosis of PNMA and PTB [12]. The results revealed that satellite lesions were more often observed in the PTB group, and the mean CT attenuation of PTB shown on the plain scan was significantly higher than that of PNMA (35.15 ± 16.00 vs. 24.00 ± 12.67 HU; *P* < 0.01). However, the enhanced value of PTB on venous scans was significantly lower than that of PNMA (13.44 ± 13.40 vs. 22.52 ± 14.00 HU; *P* = 0.02). However, the noninvasive imaging diagnostic information provided by preoperative CT images is still limited and cannot accurately differentiate PNMA from PTB. Radiomics can describe the characteristics of the lesion by high-throughput extraction of a large number of medical image features and is an emerging technology that could enhance clinical decision-making [14, 15]. Radiomics models have been indicated to be favorable for diagnosing lung nodules in clinical applications, such as distinguishing benign from malignant nodules, for the preoperative prediction of nodule type, for prognostic analysis, and for predicting the outcome of a surgery, gene expression pattern and microenvironment of tumor [16–18]. Imaging examination is one of the routine procedures of daily clinical diagnosis, so radiomics research is readily feasible. Based on these former explorations, to better satisfy the needs of precise evaluation of pathology, radiomics features were explored and used to develop a model to differentiate PNMA from PTB in our present study.

## Materials and methods

This study was performed following the Helsinki Declaration and approved by the Ethics Committee of our hospital (Ethics Committee of Hebei Medical University Fourth Affiliated Hospital, reference number: 2022KS017, data 2022.6.27). Ethical approval was obtained from our hospital. And it was approved by the Ethics Committee of our hospital (Ethics Committee of Hebei Medical University Fourth Affiliated Hospital, reference number: 2022KS017, data 2022.6.27) that waivers of consent were granted to the study subjects. As a retrospective study, the exemption from obtaining consent does not affect the rights and interests of the research participant [19]. All methods were performed in accordance with the relevant guidelines and regulations.

### Patient selection

We retrospectively analyzed patients with lung mucinous adenocarcinoma and pulmonary tuberculoma diagnosed from January 2017 to November 2022 in The Fourth Affiliated Hospital of Hebei Medical University. The inclusion criteria of patients with PNMA and PTB were as follows: (1) surgical pathology-confirmed invasive mucinous adenocarcinoma (mucinous adenocarcinoma component > 90%) and pulmonary tuberculoma; (2) the maximum diameter of the nodule was less than or equal to 3.0 cm; (3) solitary and solid nodules without calcification, which may contain cavities or vacuoles and do not exhibit a ground glass density; and (4) complete clinical and pathological data, including analyzable plain and enhanced thin-slice CT image data (1.25 mm/slice) and available CT images within 2 weeks before the pathological diagnosis. The exclusion criteria of patients were as follows: (1) multiple pulmonary nodules; (2) antitumor therapy prior to CT examination and pathological diagnosis; (3) other types of cancer or incomplete clinical and imaging data; and (4) lymph node metastases and/or distant metastases [19]. In this retrospective study, a total of 177 patients were enrolled, and their ages ranged from 20 to 81 years. The patients were randomly divided into a training group and a test group at a ratio of 7:3, including 123 patients in the training group (86 patients with PNMA, 37 patients with PTB) and 54 patients in the test group (38 patients with PNMA and 16 patients with PTB). Using retrospective data from the training group, a nomogram was developed, which was internally validated using data from the test group. Additionally, we collected a dataset (*n* = 68) from January 2017 to November 2022 in Xing Tai People’s Hospital (30 and 14 patients with PNMA and PTB, respectively) and The First Hospital of Xing Tai (16 and 8 patients with PNMA and PTB, respectively) to validate the nomogram externally. The inclusion and exclusion criteria were the same as for the development cohort. Our hospital's ethical review board approved this retrospective analysis, and the requirement for informed consent was waived.

### CT image acquisition

For all patients, contrast-enhanced chest CT scans were conducted with a 256-multidetector CT scanner (Discovery CT 750 HD Revolution, GE Medical Systems, Milwaukee, Wisconsin, USA). Before scanning, the patients were trained to breathe and hold their breath at the end of inspiration to obtain the scans. The patients were placed in the supine position with both arms raised to reduce scanning artifacts. The locational marker was the sternoclavicular joint, and the range included the thoracic inlet to the lung bases. The scanning parameters were as follows: tube voltage 120 kV, tube current 200 mAs, reconstruction layer thickness 1.25 mm, matrix ​​512 × 512, and pitch 1.2. The reconstruction algorithm adopts the lung algorithm. After the plain scan was completed, 70–90 ml of nonionic contrast agent iohexol or ioversol (300 mg·I/ml) was injected via a bolus through the cubital vein with a high-pressure syringe at a flow rate of 3 ml/s. Arterial phase and venous phase dual-phase enhanced scans were performed 30 s and 90 s after the injection of the contrast agent, respectively. The other parameters were the same as those for the plain scans. After scanning, the raw data were uploaded to a postprocessing workstation for multiplanar reconstruction (MPR) [19].

Image feature analysis was performed by two board-certified thoracic group radiologists (with 6 and 12 years of experience in chest CT imaging, respectively) who were blinded to the clinical and histological findings. The mediastinal window (window width 400 HU; window level 40 HU) and lung window (window width 1200 HU; window level − 600 HU) were set. The CT image features recorded in the image analysis were as follows: (1) primary tumor location (left and right lungs, upper, middle and lower lobes); (2) tumor size (maximum diameter), mean CT value (plain scan, venous phase), ΔCTV (the difference in the mean CT value of the venous phase and the mean CT value of the plain scan), and edge (lobular, blur); (3) internal features of the tumor: the presence or absence of cavities or vacuoles; and (4) external features of the tumor: the presence or absence of satellite lesions around the nodules. The mean CT values of the nodules on the plain scan and the venous scan were measured. The cavity and vacuole were defined as a gaseous density with a maximum diameter greater than 5 mm and less than 5 mm, respectively. The satellite lesions were defined as ≥ 1 miliary nodule surrounding each nodule (within 3.0 cm), apart from one miliary nodule distal to each nodule (possible obstructive inflammation). All CT image features were independently recorded by two radiologists, and any discrepancies in assessments were consistently resolved [19].

### Segmentation, feature extraction, and selection

The CT images were imported into the open-source software 3D-Slicer (version 5.0.2, http://www.slicer.org) and were read under the lung window (width 1500/-600 HU) and mediastinal window (width 400/40 HU) settings. The primary lesions of the patients with PNMA or PTB were selected for tumor segmentation after image acquisition. A radiologist without knowledge of the clinical data manually delineated regions of interest (ROIs) layer by layer. The tumor ROI encompassed the entire lesion as much as possible, including cavities or vacuoles within the nodules and excluding bronchi, blood vessels, and normal lung tissue. Tumor segmentation was performed on 40 patients who were randomly selected from the entire cohort for independent segmentation to assess the intraclass agreement one month later. Another radiologist repeated the independent segmentation of the selected 40 patients and evaluated the interclass agreement. Intra- and interclass correlation coefficients (ICCs) were used to assess the intraobserver and interobserver reproducibility of feature extraction.

Pyradiomics in 3D-Slicer software was used to extract the radiomics features. The detailed extraction customization was as follows: 1) Feature classes: All features; 2) Resampling and filtering: Resampled voxel size (3,3,3), LoG kernel sizes (4,5), and Wavelet-based features (√). A total of 1037 features were extracted, including 17 histogram classes, 14 form factor classes, 24 Gy level cooccurrence matrix (GLCM) classes, 16 Gy level run length matrix (GLRLM) classes, 16 Gy level size zone matrix (GLSZM) classes, 5 neighboring gray tone difference matrix (NGTDM) classes, and 14 Gy level dependence matrix (GLDM) classes.

To reduce the dimensionality of the radiomic features to the number of events, we performed three sequential steps for radiomic feature selection. First, we evaluated the interobserver agreement of radiomic features and selected features showing ICC > 0.75. For the next step, we chose radiomic features that showed statistical significance between the PNMA and PTB groups. Finally, the least absolute shrinkage and selection operator (LASSO) logistic regression model was used to choose the most useful predictive features of radiomics for the differentiation of PNMA from PTB in the training group: fivefold cross validation was performed 100 times to avoid overfitting.

### Model development

Three radiomics prediction models, logistic regression (LR), support vector machine (SVM) and random forest (RF), were applied. The best performing model was adopted, and the radiomics score (Radscore) was then computed.

At the same time, we constructed a model based on clinical and CT features for the multivariate logistic regression analysis. The clinical features included sex, age, smoking status, and diabetes history. The CT features are illustrated above.

Finally, three models, the clinical model, radiomics model and the combined model based on the clinical factors and radiomics features, were compared statistically to identify the model with the highest predictability.

### Statistical analysis

All statistical analyses were performed using R version 3.6.3 and Python version 3.7. The patients were randomly divided into a training group and a test group at a ratio of 7:3. All radiomic features were applied with Z score normalization. Baseline data were analyzed by univariate analysis using Python statsmodels 0.11.1. The chi-square test was used for categorical variables, and the t test or Mann‒Whitney U test was used for continuous variables. The factors with significant differences (*P* < 0.05) were included in the multivariate logistic regression analysis. The clinical and CT features with significant differences (*P* < 0.05) in the multivariate analysis results were selected to construct the clinical prediction model [19].

The main evaluation indicators were the area under the ROC curve, accuracy, sensitivity, specificity, positive predictive value and negative predictive value. Decision curve analysis (DCA) was used to calculate the clinical impact of the three models [19].

## Results

### Patient characteristics

In all enrolled patients, there was no significant difference in age between the two groups, and the median ages were 59 and 62 years in the patients with PTB and PNMA, respectively. The proportion of female patients was higher in the patients with PNMA than in the patients with PTB (50.00% and 24.42%, *P* = 0.004). Smoking history was more prevalent in patients with PTB than in patients with PNMA (62.26% and 34.68%, *P* < 0.001). There was a higher proportion of patients with a history of diabetes in the PTB group than in the PNMA group. In the PNMA group and the PTB group, the proportion of lesions located in the lower lobe was 66.94% and 22.64%, respectively. Between the PNMA and PTB groups, the proportion of satellite lesions on CT lung window images was statistically significant (50.94% and 4.03%, respectively *P* < 0.001). The proportion of cavities or vacuoles was higher in the patients with PNMA than in the patients with PTB (52.42% and 24.53%, respectively). *P* < 0.001). The mean CT value on the plain scan of PNMA was significantly lower than that of PTB (17.00 HU vs. 31.00 HU; *P* < 0.001). The ΔCTV of PNMA was significantly higher than that of PTB (25.49 HU vs. 4.00 HU; *P* < 0.001). The best cost values of plain CT value and ΔCTV were 29 HU and 12 HU, respectively. The characteristics of the patients in the training and testing cohorts are shown in detail in Table 1 [1].

**Table 1:** Clinical characteristics of the patients

Characters	Training cohort			Testing cohort		
	PTB	PNMA	*p*	PTB	PNMA	*p*
Gender Female	25	50	0.078	14	12	0.006
Male	9	39		5	23	
Smoking No	10	58	< 0.001	10	23	0.346
Yes	24	31		9	12	
Diabetes No	25	79	0.037	11	30	0.022
Yes	9	10		8	5	
Edge clear NO	1	13	0.068	3	5	0.882
Yes	33	76		16	30	
Lobul No	3	34	0.001	1	13	0.011
Yes	31	55		18	22	
Spicul No	28	63	0.191	12	24	0.687
Yes	6	26		7	11	
Satellite No	16	85	< 0.001	10	34	< 0.001
Yes	18	4		9	1	
Cavity No	24	43	0.027	16	16	0.006
Yes	10	46		3	19	
Lower lobe No	26	31	< 0.001	15	10	< 0.001
Yes	8	58		4	25	
△CTA, median[IQR]	2.000[0.602,11.000]	18.000[8.000,40.000]	< 0.001	1.000[1.000,3.000]	25.000[12.000,46.000]	< 0.001
△CTV, median[IQR]	5.000[2.000,15.000]	23.000[14.000,38.489]	< 0.001	2.000[1.000,6.000]	37.795[16.000,60.712]	< 0.001
Plain, median[IQR]	30.950[21.000,41.000]	12.000[-46.288,27.000]	< 0.001	32.000[23.000,60.000]	21.000[-40.000,26.436]	< 0.001
Diameter, median[IQR]	2.000[1.600,2.400]	1.700[1.200,2.300]	0.131	1.700[1.300,2.000]	1.400[1.100,2.000]	0.224
Age, median[IQR]	56.000[51.000,64.000]	62.000[56.000,67.000]	0.036	57.474 ± 12.991	59.657 ± 10.370	0.511

### Feature selection and clinical model construction

The multivariate analysis showed that there were significant differences between PNMA and PTB in smoking, diabetes history, lesion located in the lower lobe, satellite lesions, cavity or vacuole, plain CT value and ΔCTV. The clinical prediction model was established by logistic regression based on the seven features. The AUC values of the clinical models in differentiating PNMA from tuberculoma were 0.918 and 0.888 in the training group and test group, respectively. See Table 2 for details.

**Table 2:** Multivariate analysis to identify significant factors for PTB and PNMA

Predictor	*p*	Odds Ratio	Lower	Upper
△CTV	0.006	1.026	1.008	1.047
Plain	0.004	0.978	0.961	0.991
Diabetes	0.132	0.415	0.129	1.301
Smoking	0.127	0.464	0.17	1.245
Satellite	0.0	0.076	0.017	0.272
Cavity	0.011	4.399	1.493	15.097
Lower lobe	0.014	3.465	1.299	9.625

### Radiomics feature selection and model construction

**Fig. 1:**
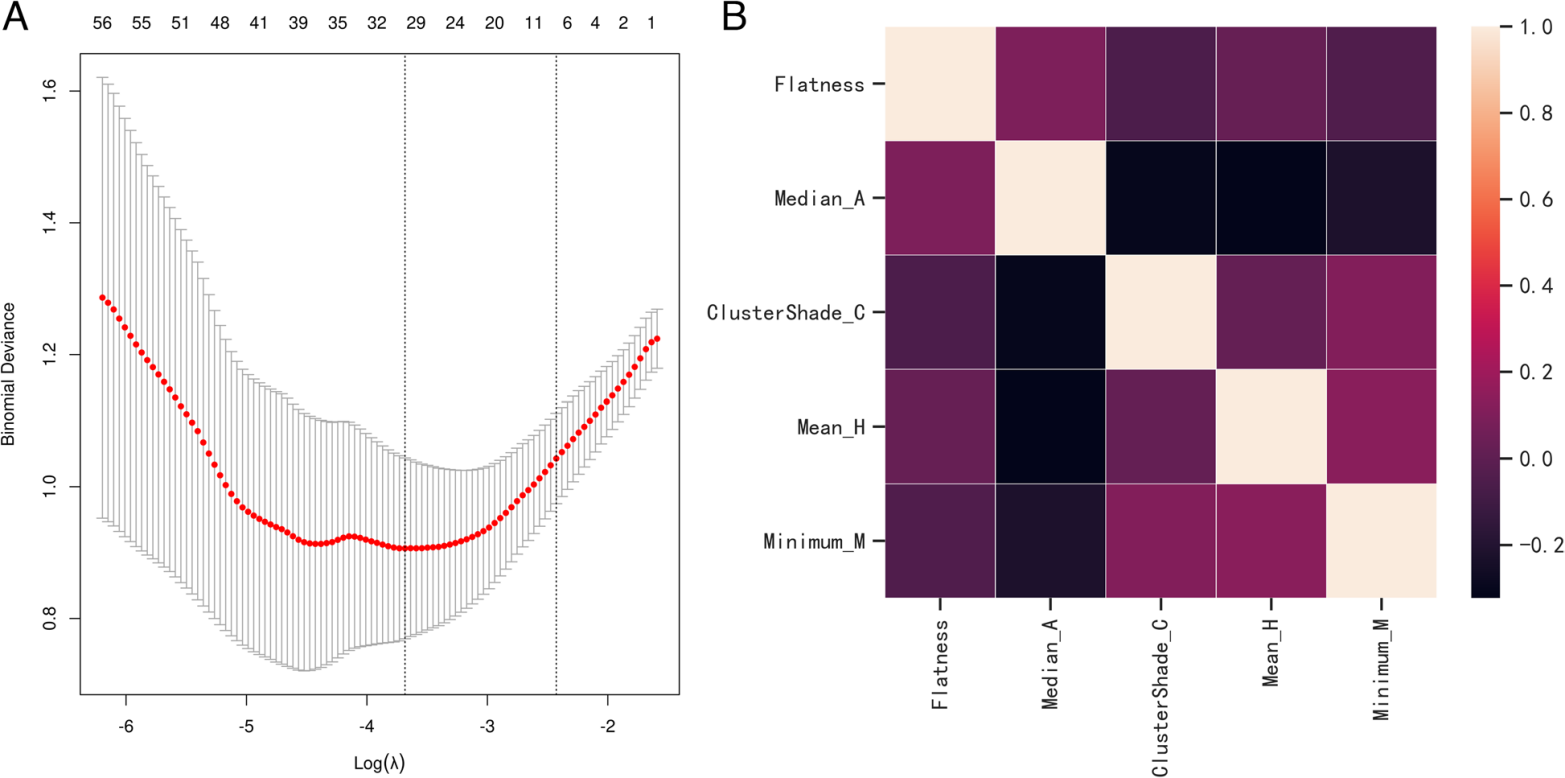
Radiomics feature selection

To eliminate redundant features, 688 features that showed no significant difference between PNMA and PTB and 2 highly correlated features with ICC values less than 0.75 were excluded. After screening out the redundant features by LASSO and correlation analysis, the five most robust radiomics features (including flatness, cluster shade, minimum, median and skewness) were retained. The relationships of the five radiomics features were shown to be significantly low (Fig. 1).

**Fig. 2:**
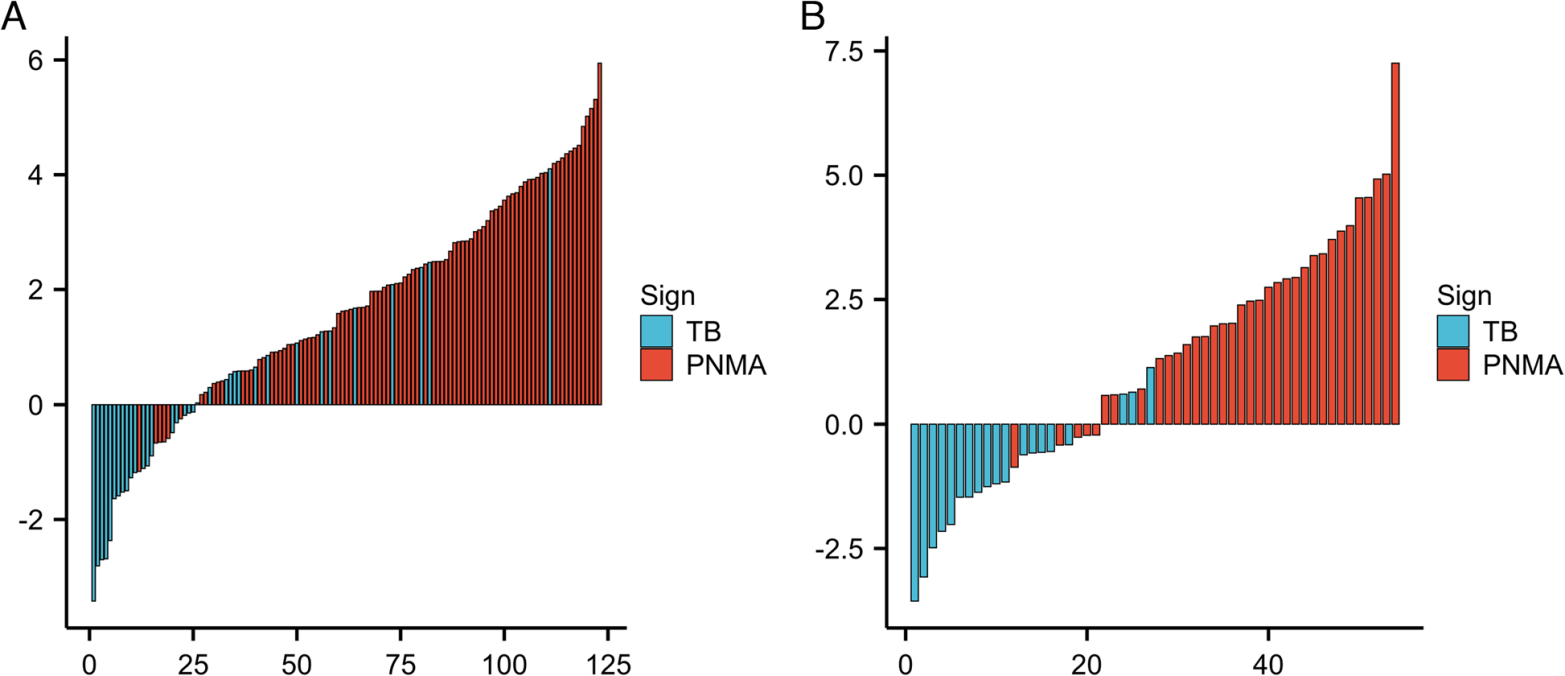
Bar charts of the Radscore for each patient in the training cohort (**A**) and testing cohort (**B**)

The machine learning models logistic regression (LR), support vector machine (SVM) and random forest (RF) were developed based on the five radiomics features. The model established by the LR method had the best performance, and the AUC values in the training group and test group were 0.840 and 0.960, respectively (Table 3 and Fig. 4). The Radscore for each patient was then calculated by selected features weighted by their respective coefficients in the LR model, which can be expressed as follows: Radscore = 1.284 + 2.169*Flatness + 0.911*Median-0.613*Cluster Shade-0.482*Mean-0.941* Minimum. The Radscore for each patient in the training group and test group is shown in Fig. 2.

**Table 3:** Diagnostic performance of the prediction models

Model	Training cohort			Testing cohort			External validation		
	AUC	SEN	SPE	AUC	SEN	SPE	AUC	SEN	SPE
RADS	0.840	0.809	0.765	0.960	0.943	0.842	0.840	0.773	0.810
Clinical	0.920	0.820	0.912	0.950	0.914	0.947	0.890	0.773	0.857
Comb	0.940	0.899	0.912	0.990	0.943	1.000	0.960	0.864	0.952

### Combined model construction and validation of performance

**Fig. 3:**
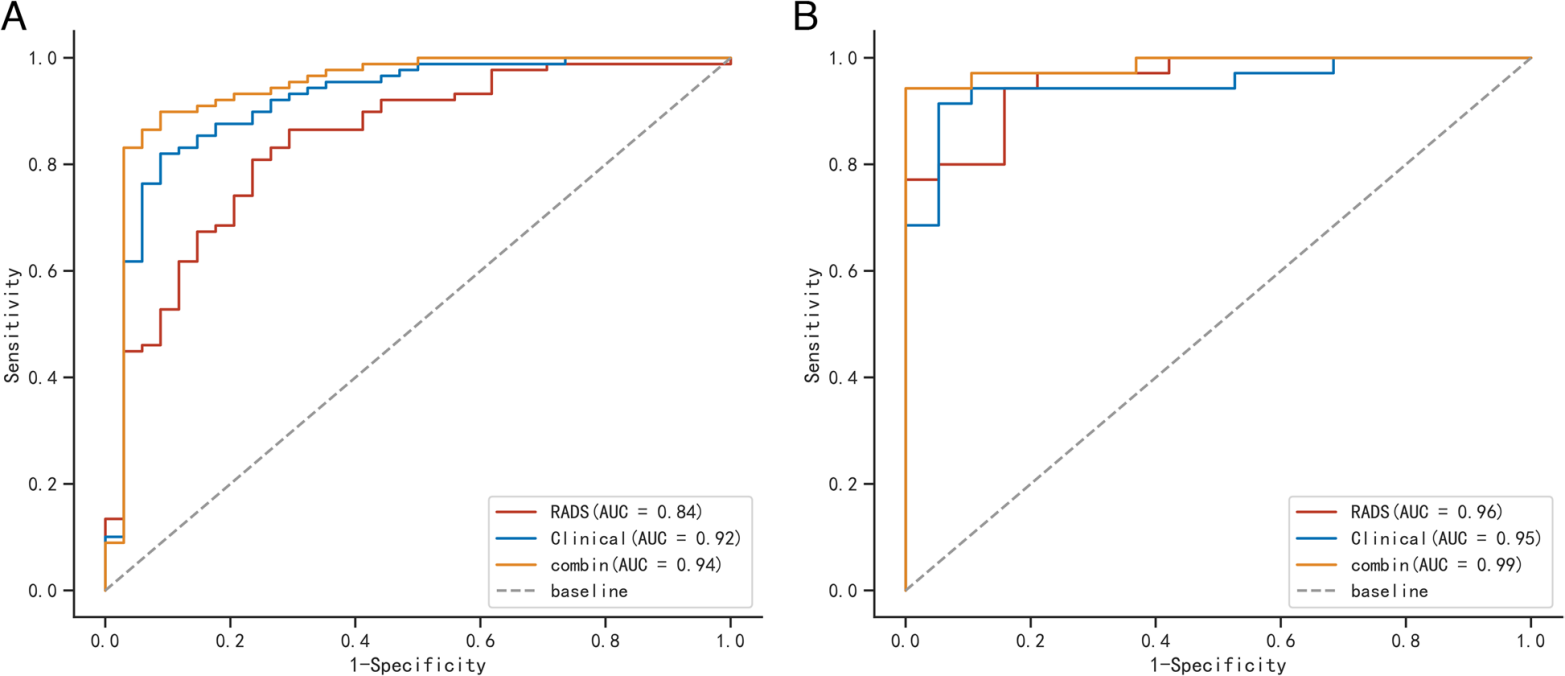
Comparison of receiver operating characteristic (ROC) curves among the clinical model, radiomics model and combined model in the training cohorts (**A**) and testing cohorts (**B**)

**Fig. 4:**
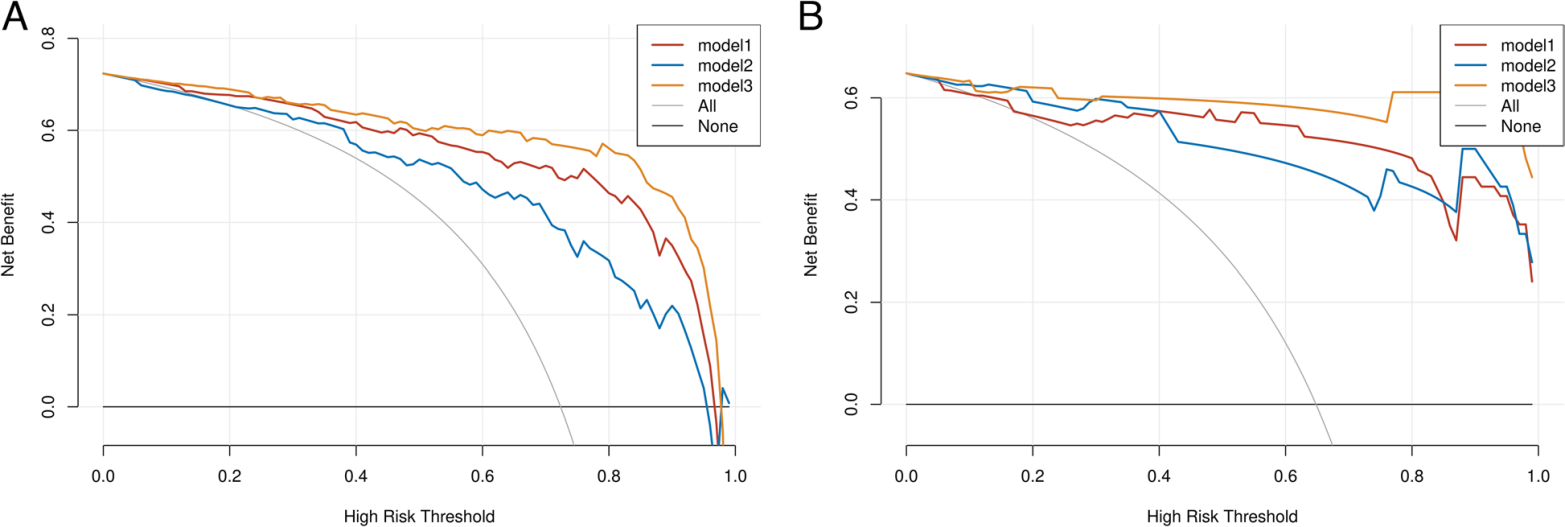
Decision curve analyses for the radiomics-clinical model compared with the radiomics model and clinical model in the training cohort (**A**) and the testing cohort (**B**)

**Fig. 5:**
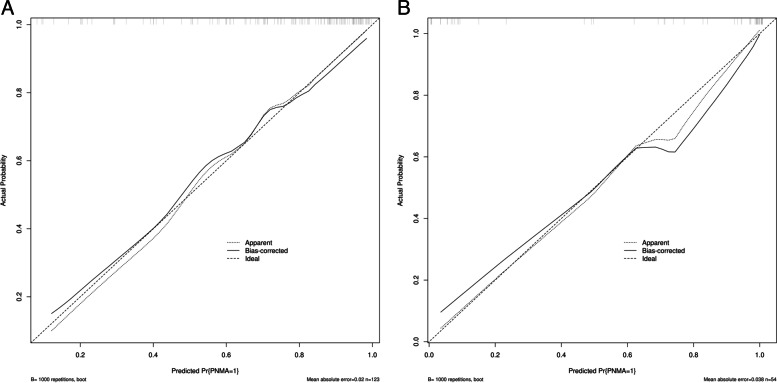
Calibration plot of the combined model in the training group

Logistic regression was performed to establish a combined model based on seven clinical features and five radiomics features. The results revealed that the combined model and radiomics model were superior to the clinical model and radiomics model, with ROC-AUCs of the combined model, radiomics model and clinical model of 0.940 and 0.990, 0.840 and 0.960 and 0.920 and 0.950 in the training and test groups, respectively (Table 3 and Fig. 3), respectively. The combined model had very high sensitivity and specificity, 0.899 and 0.912, 0.943 and 1.00 in the training and test groups, respectively (Table 3). The decision curve analyses revealed that when the probability of the threshold was over 0%, the net benefits of the combined model for preoperative differentiation of PNMA from PTB were higher than those of the clinical model and radiomics model in both the training group and test group (Fig. 4). The Brier scores of the combined model were 0.132 and 0.068 in the training group and test group, respectively, and the calibration plots are shown in Fig. 5.

### External validation of the three models

The external validation group included 68 patients in the final analysis. Table 1 shows the baseline characteristics of the external group. The AUC values of the clinical model, radiomics model and combined model in differentiating PNMA from PTB were 0.890, 0.840 and 0.960 in the external validation group, respectively (Table 3 and Supplementary Fig. 1).

## Discussion

According to this retrospective study, there were some significant differences between PNMA and PTB patients with solitary pulmonary solid nodules in terms of qualitative and quantitative clinical characteristics. We selected seven clinical variables to build our clinical prediction model to differentiate PNMA from PTB, including smoking history, diabetes history, lesion located in the lower lobe, satellite lesions, cavity or vacuole, plain CT value and ΔCTV [19]. A total of five radiomic features were selected to build the radiomic prediction model, including flatness, cluster shade, minimum, median and skewness. The clinical-radiomics combined model, consisting of the above seven clinical features and five radiomics parameters, demonstrated good predictive ability in both the training and validation sets. Moreover, there were statistically significant differences among the clinical model, radiomics model, and combined clinical-radiomics model. The clinical-radiomics combined model was better than both single models. An external validation group of 68 patients (46 and 22 patients with PNMA and PTB, respectively) was successfully used to validate the clinical-radiomics combined model.

In this study, smoking was an independent factor to distinguish PNMA and PTB. In a previous study, cigarette smoking increases not only the risk of progression to active pulmonary tuberculosis disease but also the risk of acquiring new tuberculosis disease infection [20]. According to another study, there was also a significant linear dose–response relationship between QFT positivity and smoking duration (by years) among ever smokers (*p* < 0.001) [21]. This can be explained by the result that lysosomal storage also hinders alveolar macrophage migration to mycobacteria in the lung, suggesting a mechanism for the observed susceptibility of smokers to new tuberculosis disease infection. According to a study specifically relating smoking history to the mucinous phenotype, mucinous adenocarcinoma had little relationship to smoking history [22]. Consequently, we found that smoking history was more prevalent in PTB than in PNMA. Previous studies have shown that diabetes patients' immune systems are impaired, making them more susceptible to tuberculosis [23, 24]. Diabetes mellitus did not significantly affect lung cancer risk in patients with diabetes mellitus (RR: 1.10; 95% CI: 0.99–1.23; *P* = 0.087) [25]. The present study revealed a similar result: more than half of the PTB patients had diabetes complications, whereas only a few patients with PNMA were complicated with diabetes.

In histology, PNMA is characterized by mucin-rich tumor cells, fibrosis, with central fibrosis and alveolar spaces filled with mucin [26, 27]. PTB is composed of fibrous tissue containing caseous necrotic tissue. It is very easy to misdiagnose one as the other since both are low-density on plain scans and their morphological characteristics are very similar. Based on our multivariate logistic regression analysis, we found that PNMA had a higher tendency to occur in the lower lobe than PTB, whereas patients with PTB showed obvious upper lobe distribution preponderance (73.58%). Our findings were consistent with the reports by Xu [28] and Zhang et al. [29]. A possible explanation for the difference is that the tumor cells of PNMA originate from columnar epithelial cells or goblet cells. The cancer cells are relatively well-differentiated, and they can produce more mucus in response to gravity. Therefore, PNMA was much more frequently found in the lower lobe. PTB usually occurs in the apical or posterior segment of the upper lobes and lingular segment on both lower lobes. Tuberculosis bacilli are more likely to colonize and cause disease in cultures with poor blood circulation (the number of macrophages is small) and ventilation (bacteria easily survive). The “satellite lesions” usually refer to small discrete shadows in the immediate vicinity of the main lesion. As a characteristic manifestation of tuberculosis, it has become widely accepted. The pathological basis may be the spreading focus and fibroproliferative focus around the tuberculosis lesion. 26 of the 53 PTB patients had satellite lesions, with a ratio of 1:2. In contrast, 5 of the 124 PNMA had satellite lesions, with a ratio of 1:25. Compared to PNMA, PTB has a high prevalence of satellite lesions, as reported previously [29, 30]. Cavities or vacuoles were found in more than half of the patients with PNMA, but in only a quarter of the patients with PTB. Cavities appear in PNMA due to incomplete obstruction of the bronchioles by the build-up of mucus, resulting in alveolar hyperventilation. On the other hand, vacuoles may be caused by internal necrosis of the tumor, and the necrosis is eliminated through the bronchus. Therefore, cavities or vacuoles were much more common in PNMA.

CT scanning, especially CT dynamic contrast-enhanced scanning, is a valuable tool in the diagnosis of lung cancer and tuberculosis. The CT features of lung adenocarcinoma and pulmonary tuberculoma have received much attention in the literature, but PNMA and PTB have received fewer reports [12, 29, 30]. As a result of this study, we determined that the mean CT value of PTB on plain scan was 31.00 HU and that the CT value of PNMA was 17.00 HU, whereas the ΔCTV was 25.49 HU in the PNMA group and 4 HU in the PTB group. The CT value of the PTB group on plain scan was significantly greater than that of the PNMA group, whereas the ΔCTV in the PTB group was lower than that in the PNMA group, which is inconsistent with a former report [28]. As PNMA and PTB have different pathologic bases, this may explain the difference. The PNMA contains mucin-rich tumor cells, fibrous tissues, and alveolar spaces filled with mucin proteins, resulting in a low CT value. PTB is composed of fibrous tissue that contains caseous necrotic tissue with low density, but calcification can easily occur. There are some calcifications that are fine sand and scattered, which results in a higher CT value. PNMA exhibited a complex pattern of enhancement based on the amount of solid component, fibrous tissue, and mucin present. Additionally, papillary or alveolar components within PNMA increased the difference between CT values. The center of tuberculoma is necrotic tissue with no blood supply, the periphery is a capsule, and the inner layer is granulation tissue with blood supply. As a result, enhancement is either nonenhanced, annular, or in other forms, depending on the degree of necrosis in the caseous region and the presence of granulation tissue.

To explore a much more effective method to differentiate PNMA from PTB, we extracted five independent radiomic features associated with PNMA and PTB, including flatness, cluster shade, minimum, median and skewness. These parameters belong to the Form Factor Parameters, Texture Parameters, and Histogram Parameters. Flatness is a form factor parameter that is independent of the gray-level intensity distribution in the ROI. Cluster Shade, as one of the Texture Parameters, is the task of grouping a group of objects so that objects in the same group (cluster shade) are more similar to each other (in a sense) than objects in other groups (cluster shade). The larger the Cluster Shade value, the more asymmetrical it is. The minimum belongs to the histogram parameter, which represents the minimum pixel value of an image (of the lesion). The median is also a histogram parameter that represents the median pixel value of an image (of the lesion). Another histogram parameter is skewness, which reflects the degree of asymmetry in the histogram distribution, and if the predictive value has been effective, the absolute values of the skewness would have been higher. All features above were obtained via the conversion of images to higher-dimensional data. They allowed high-throughput mining of quantitative imaging features from general medical images, followed by an automated analysis to assist clinical decision-making. Previous studies revealed that radiomics features from CT could preoperatively differentiate lung adenocarcinoma from lung tuberculoma in patients with pulmonary solitary solid nodules and could also distinguish adenocarcinomas from granulomas. To our knowledge, this is the first study to differentiate PNMA from PTB based on radiomic features. In this study, the model based on these radiomic features also illustrated an effective role in preoperatively differentiating PNMA from PTB. According to our ROC analyses, the AUC values for the radiomics model were 0.840 and 0.960 in the training and test groups, respectively. The clinical-radiomics combined model (ROC-AUC: 0.940–0.990) was significantly better than the clinical model and radiomics model. Furthermore, the decision curve analysis also demonstrated that the combined model performed significantly better than the clinical model and radiomic model in predicting outcomes. Decision curve analysis offers important information beyond the standard performance metrics of discrimination and calibration and could be used to evaluate the clinical impact, indicating that there was a higher chance of success. In this study, not only an internal validation was carried out, but an external validation was also carried out on this basis. At the same time, the ROC curve analysis showed that the AUC of the clinical-radiomics combined model was greater than 0.9000 in both the internal validation and external validation. This suggested that the clinical-radiomics combined model had a good discrimination degree and a strong ability to distinguish PNMA from PTB.

Our study had several limitations that must be considered. First, it was a retrospective study with a relatively small sample size. Second, due to the study's inclusion of only patients who had pathologic results after surgery, selection bias cannot be ignored. Additionally, only PNMA and PTB were observed without calcification, so the results should be interpreted cautiously. Third, our study only evaluated the relationship between PNMA and PTB, and other types of lung nodules need to be further explored, such as lung squamous cell carcinoma and other benign granulomatous lesions. To guide clinical practice, the model will be validated in a multicenter, prospective, large-scale study in the future and further optimized.

In conclusion, in our present study, we established a model to differentiate PNMA from PTB by using preoperative clinicopathological features, radiomic features, and clinical-radiomic features for the first time. The clinical-radiomic model established that we established also showed good predictive value and had potential value in clinical practice.

## Supplementary Information

**Additional file 1. **

**Additional file 2. **

## Data Availability

The datasets generated and analyzed during the current study are not publicly available because the dataset will be further studied to publish other works but are available from the corresponding author on reasonable request.
