# Supplementary figures and images for: Radiomics nomogram for preoperative differentiation of pulmonary mucinous adenocarcinoma from tuberculoma in solitary pulmonary solid nodules
Source: BMC Cancer. 2023 Mar 21;23:261. doi: 10.1186/s12885-023-10734-4 (PMC10029225; doi:10.1186/s12885-023-10734-4)

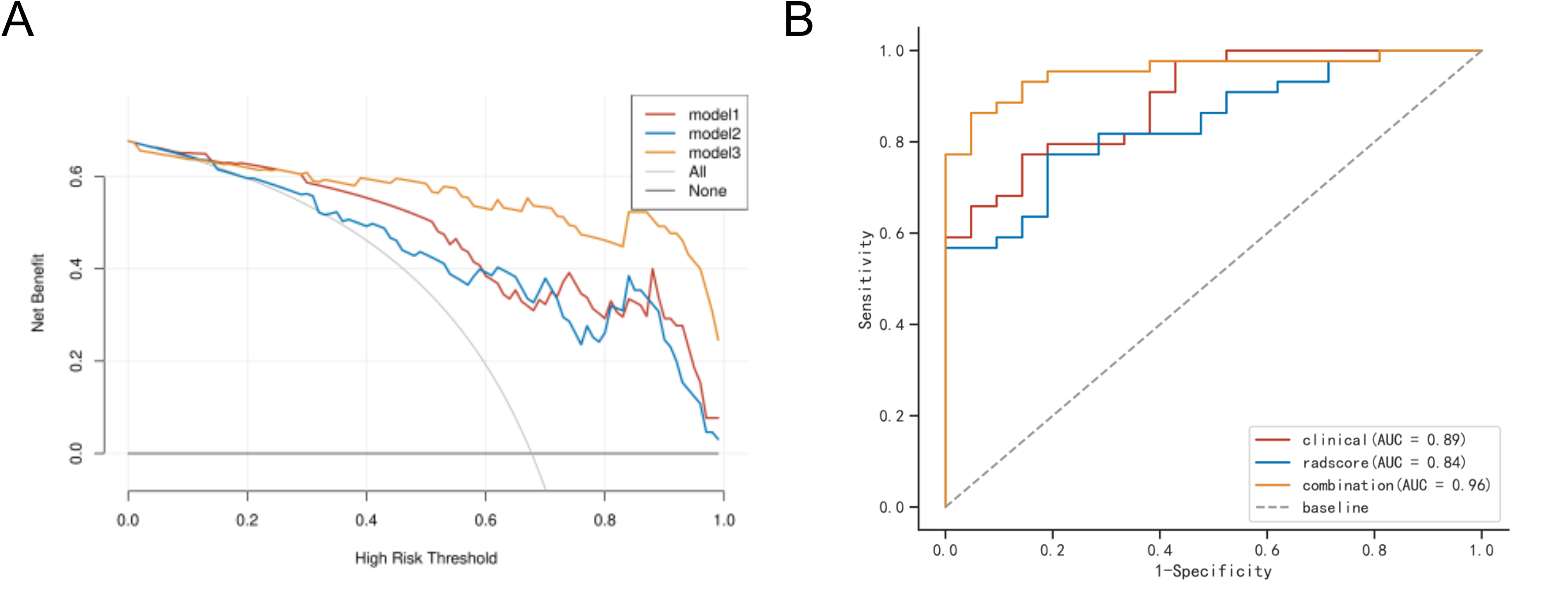

Supplement: Supplementary file 1 — Additional file 1. [file 12885_2023_10734_MOESM1_ESM.tiff]
