# Supplementary material for: Radiomics nomogram for preoperative differentiation of pulmonary mucinous adenocarcinoma from tuberculoma in solitary pulmonary solid nodules
Source: BMC Cancer. 2023 Mar 21;23:261. doi: 10.1186/s12885-023-10734-4 (PMC10029225; doi:10.1186/s12885-023-10734-4)
Supplement: Supplementary file 2 — Additional file 2. [file 12885_2023_10734_MOESM2_ESM.docx]

**Table 1 Characteristics of the patients in the external validation group**

| Characters | PTB | PNMA | p |
| --- | --- | --- | --- |
| Gender Female | 17(80.952) | 13(29.545) | <0.001 |
| Male | 4(19.048) | 31(70.455) |  |
| Smoking No | 8(38.095) | 31(70.455) | 0.013 |
| Yes | 13(61.905) | 13(29.545) |  |
| Diabetes No | 13(61.905) | 37(84.091) | 0.047 |
| Yes | 8(38.095) | 7(15.909) |  |
| Lobul No | 5(23.810) | 16(36.364) | 0.311 |
| Yes | 16(76.190) | 28(63.636) |  |
| Spicul No | 16(76.190) | 32(72.727) | 0.766 |
| Yes | 5(23.810) | 12(27.273) |  |
| Cavity No | 16(76.190) | 22(50.000) | 0.045 |
| Yes | 5(23.810) | 22(50.000) |  |
| Edge clear NO | 6(28.571) | 6(13.636) | 0.147 |
| Yes | 15(71.429) | 38(86.364) |  |
| Satellite No | 8(38.095) | 43(97.727) | <0.001 |
| Yes | 13(61.905) | 1(2.273) |  |
| Lower lobe No | 15(71.429) | 12(27.273) | <0.001 |
| Yes | 6(28.571) | 32(72.727) |  |
| △CTV, median[IQR] | 10.429±8.162 | 28.500±10.062 | 0.001 |
| Plain, median[IQR] | 31.571±7.632 | -16.933±97.513 | 0.012 |
| Diameter, median[IQR] | 2.300[2.000,2.600] | 1.400[1.100,2.000] | <0.001 |
| Age, median[IQR] | 56.429±13.168 | 60.750±9.982 | 0.154 |
